# Evaluation of Aptamer Fluorescence Microscopy in the Diagnosis of Pulmonary Tuberculosis

**DOI:** 10.1128/spectrum.02602-21

**Published:** 2022-06-14

**Authors:** Ruijuan Zheng, Feifan Xu, Xiaochen Huang, Jie Wang, Yonghong Feng, Jin Huang, Lianhua Qin

**Affiliations:** a Shanghai Key Laboratory of Tuberculosis, Shanghai Pulmonary Hospital, Tongji University School of Medicine, Shanghai, China; b Department of Clinical Laboratory, The Sixth People’s Hospital of Nantong, Nantong, Jiangsu, China; c Department of Biochemistry and Molecular Biology, Key Laboratory of Environmental Pollution Monitoring and Disease Control of Ministry of Education, Guizhou Medical University, Guiyang, Guizhou, China; Children's Hospital Los Angeles, University of Southern California

**Keywords:** aptamer, *Mycobacterium tuberculosis*, sputum sample, microscopy, tuberculosis diagnosis

## Abstract

Sputum smear microscopy for tuberculosis diagnosis has stood the test of time. However, due to its low sensitivity, the positive detection rate for tuberculosis in clinical specimens is not high. To improve the sensitivity of microscopic observation in Mycobacterium tuberculosis (MTB) detection, we developed the MTB-specific aptamer MA1. To further improve the binding reactivity of MA1 with MTB, we constructed a new derivative aptamer with a pocket-stem-loop-structure, MA1-39, and identified it to have high binding reactivity with the MTB reference strain. We developed an aptamer fluorescence microscopy test for MTB based on MA1-39 and evaluated its feasibility for diagnosing pulmonary tuberculosis. Among 56 tested strains, MA1-39 was proven to effectively discriminate MTB from the control strains, including 12 non-tuberculosis mycobacterial (NTM) reference strains, 6 NTM isolates, and 7 other bacteria. Next, this approach was applied to 169 clinical sputum samples from suspected tuberculosis patients and non-tuberculosis controls. Molecular tests together with both clinical and bacteriological identification were used as a protocol to evaluate the efficacy of aptamer detection. Compared with the traditional acid-fast staining light microscope, the aptamer fluorescence microscope showed a higher detection rate for MTB in clinical specimens (48.8% versus 32.6%), and the specificities of the two techniques had almost no significant difference (90.4% versus 94%). In addition, aptamer fluorescence microscopy showed the same positive predictive value (PPV) as staining (84% versus 84.9%), but a higher negative predictive value (NPV; 63% versus 57.3%). In conclusion, the newly established aptamer fluorescence microscopy approach is likely to be a feasible method for microbiological diagnosis of tuberculosis.

**IMPORTANCE** We established an aptamer fluorescence microscopy approach for rapid detection of MTB in clinical sputum samples. The use of aptamer probes was proven to significantly increase the sensitivity of sputum smear microscopy. In resource-limited countries, microscopy is currently a fast, simple, and very common test method in many laboratories, and it will remain the primary means of microbiological diagnosis of tuberculosis in the foreseeable future. Improving detection techniques can further enhance the clinical application value of this ancient diagnostic method. Since aptamer fluorescence microscopy can provide rapid and sensitive results, it may be a feasible and useful method in resource-limited settings.

## INTRODUCTION

Tuberculosis (TB), caused by the bacillus Mycobacterium tuberculosis (MTB), poses a major health security threat and causes considerable morbidity and mortality worldwide (1). About a quarter of the world population is infected with MTB (1). In 2020, there were an estimated 1.3 million deaths globally due to TB disease (1). Early and accurate detection of pathogenic mycobacteria in clinical samples is a critical step in the management and control of TB. Delayed diagnosis can increase the time spent on improper therapies and prolong the period of transmissibility. Bacteriological tests, including acid-fast staining methodologies paired with mycobacterial cultures, remain the “gold standard” method to specifically diagnose mycobacteria. Rapid molecular tests have been recommended for use as initial diagnostic tests for TB by the WHO, but their high cost is likely to impede their implementation in many laboratories (2, 3). Even today, acid-fast staining microscopy of sputum specimens is still widely used for TB diagnosis in many developing countries due to its cost effectiveness and rapid turnaround time (4–9). However, its low and variable sensitivity (range = 20% to 80%) is a major problem (7–9). In fact, the WHO reports that only 28% of MTB cases are smear-positive with conventional acid-fast staining techniques (9). This issue alone highlights the need for more sensitive microscopic observation methods for MTB detection. Moreover, because all mycobacterial species test positive in acid-fast staining, it is difficult to distinguish MTB from non-tuberculosis mycobacterial (NTM) species based on conventional microscopy. Thus, accurate recognition of MTB is also needed to improve the performance of sputum-smear microscopy. Recently, antibodies, aptamers, and peptides have been used as recognition molecules for pathogen identification (10, 11). Among these, aptamers have attracted considerable attention (11–13).

Aptamers, obtained by SELEX (Systematic Evolution of Ligands by EXponential enrichment), are short single-stranded DNA (ssDNA) or single-stranded RNA (ssRNA) molecules with a length of 25 to 100 bases, and can specifically bind to targets by forming stable three-dimensional conformations. When these aptamers are combined using different transduction methods, including chemiluminescence, electrochemistry, and fluorescence, they can serve as detection molecules for the specific identification of pathogens (12–16). Currently, a novel fluorescent biosensor based on aptamers has been developed and shown clinical application potential for the rapid detection of Escherichia coli in complex samples (15). Moreover, a fluorescein amidite (FAM)-labeled aptamer has been used as a fluorescence indicator for detection of Salmonella Typhimurium (16).

In our previous study, we obtained MTB-specific aptamers by whole-cell SELEX (17). A dominant aptamer with a high binding affinity, MA1, was generated and proved to efficiently capture or discriminate between MTB species (17). To further improve the binding reactivity of MA1 with MTB and evaluate its potential application for MTB detection in clinical complex samples, in this study we constructed derived aptamers of MA1 (MA1-23, MA1-39, and MA1-55) and performed clinical sample detection. In recent years, fluorescence microscopy has shown the potential to improve microscope performance (18, 19). In 2010, the WHO recommended that fluorescence microscopy be phased in as an alternative to light microscopy to improve the sensitivity of sputum-smear microscopy (20). Here, we further developed fluorescence microscopy observation based on this aptamer to improve the performance of sputum-smear microscopy and evaluate its feasibility for diagnosing pulmonary TB.

## RESULTS

### Pocket-stem-loop-structured aptamer.

**FIG 1:**
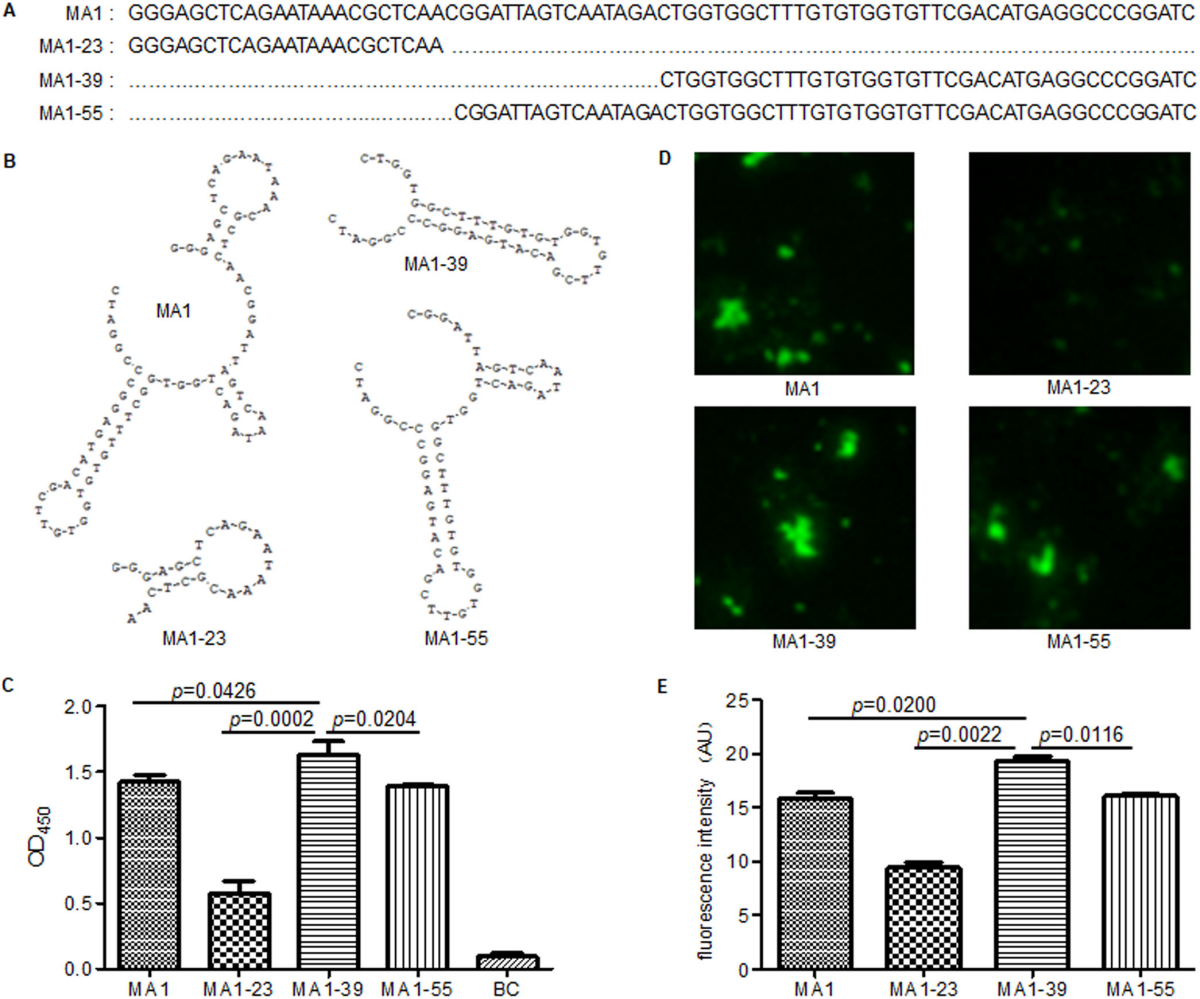
Mycobacterium tuberculosis (MTB)-specific single-stranded DNA (ssDNA) aptamer MA1 and its derived aptamers. (A) Sequences of MA1 (78-nt) and its derived aptamers MA1-23 (23-nt), MA1-39 (39-nt), and MA1-55 (55-nt). (B) Secondary structures of MA1 and its derived aptamers. (C) Binding reactivity of aptamers with H37Rv determined using an enzyme-linked immunosorbent assay. (D) Binding reactivity of aptamers with H37Rv determined by direct fluorescence microscopy observation (×20). (E) Fluorescence intensity assessed by computer-assisted analysis using Image J. Significance level was set at *P* < 0.05. BC, blank control; AU, arbitrary units.

The sequence and secondary structure of MTB-specific aptamer MA1 and its derived aptamers are shown in Fig. 1A and B. Their respective affinities against H37Rv were tested by an enzyme-linked immunosorbent assay. The results showed that the MA1-39 aptamer, with a pocket-stem-loop-structure, had the highest binding-reactivity with H37Rv. The MA1-23 aptamer, with only a pocket structure, showed a lower apparent reactivity with H37Rv; while MA1-55, with a similar structure to that of the original aptamer MA1, had similar binding reactivity with H37Rv to MA1 (Fig. 1C). All these aptamers were also labeled by fluorescein isothiocyanate (FITC) to test their binding reactivity with H37Rv using fluorescence microscopy (Fig. 1D). The fluorescence intensity of every aptamer was assessed by computer-assisted analysis using Image J (Fig. 1E). The MA1-39 aptamer had the highest fluorescence intensity (mean = 19.26 arbitrary units [AU]), followed by MA1-55 (16.04 AU) and MA1-23 (9.40 AU). The fluorescence intensity of the original aptamer MA1 was 15.84 AU.

**FIG 2:**
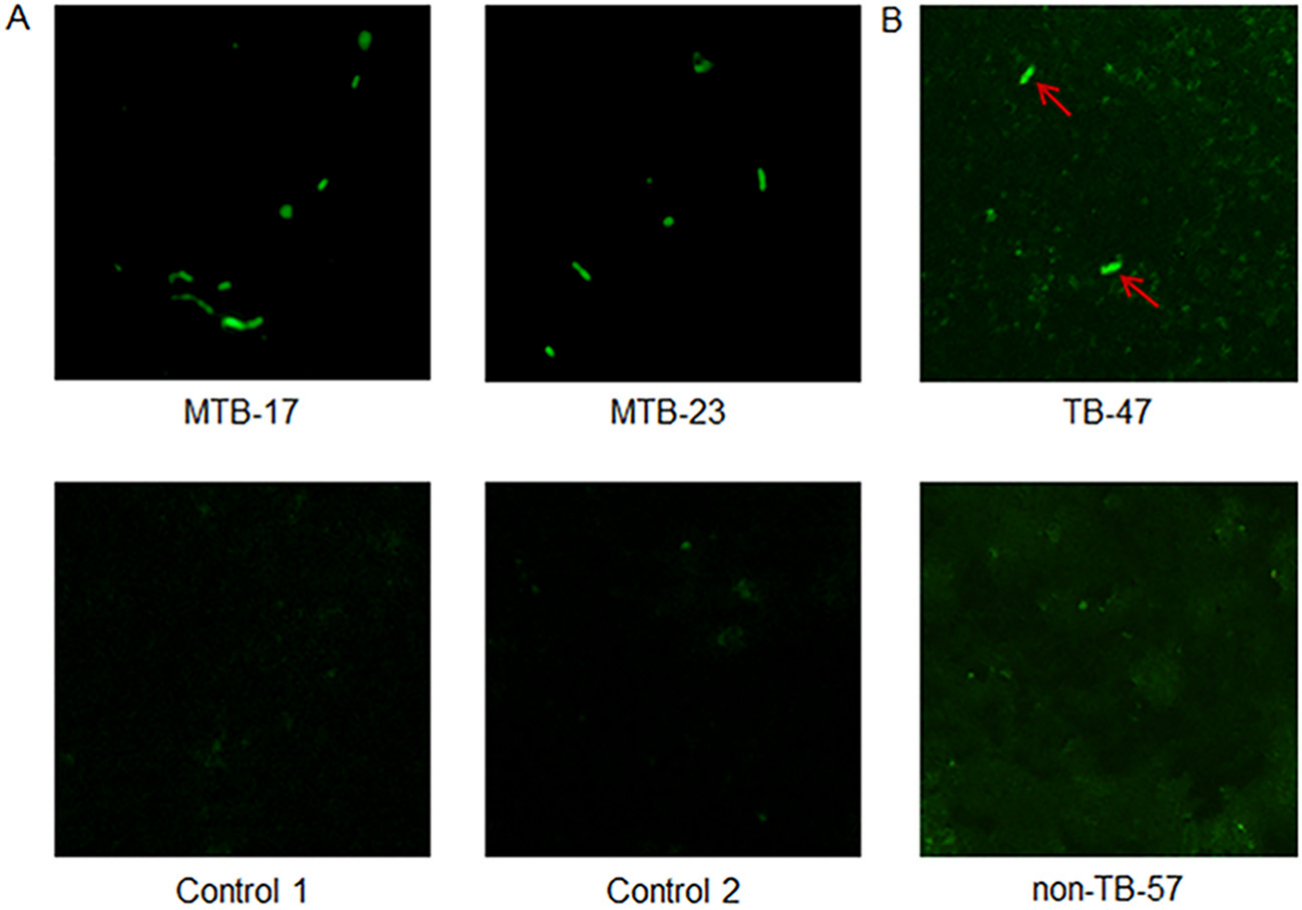
The identification of MTB strains by direct MA1-39 fluorescence-microscopy observation (×40). (A) Test in cultured strains. MTB-17 and MTB-23 are two isolates from MTB. Controls 1 and 2 are isolates from non-tuberculosis mycobacteria (NTM) and non-mycobacteria, respectively. (B) Detection in clinical sputum samples. TB-47 is the sputum sample from one TB patient, non-TB-57 is the sputum sample from one non-TB patient.

The efficiency of MA1-39 in the detection of MTB was further tested against 13 reference strains and 43 isolates (Fig. 2A, Table 1). A 570-bp DNA fragment was successfully amplified by 16SrRNA gene primers in all mycobacteria. The MTB reference strain H37Rv and 30 isolates were all positive by IS6110 PCR amplification and MA1-39 fluorescence microscopy observation. All NTM reference strains (*n* = 12) and isolates (*n* = 6) tested negative by IS6110 amplification. MA1-39 showed no significant cross-reactivity with almost all tested NTM, but still had low apparent cross-reactivity with M. marinum, just like its original aptamer MA1 (17). All seven non-mycobacterial bacteria were negative by16SrRNA and IS6110-PCR amplification and by MA1-39 fluorescence-microscopy observation. FITC-labeled MA1-39 effectively discriminated MTB from NTM and non-mycobacterial bacteria.

**TABLE 1:** Efficiency of MA1-39 for M. tuberculosis identification, evaluated by 55 tested strains

Group	No. of strains	Test			Organism
		16sRNA	IS6110	MA1-39	
I	31	+	+	+	M. tuberculosis
					
II	3	+	−	−	M. intracellulare
	4	+	−	−	M. abscessus
	1	+	−	−	*M. avium*
	2	+	−	−	M. kansasii
	1	+	−	−	M. gordonae
	1	+	−	−	*M. gilvum*
	1	+	−	−	*M. aurum*
	1	+	−	+	M. marinum
	1	+	−	−	M. smegmatis
	1	+	−	−	*M. aichiense*
	1	+	−	−	*M. phlei*
	1	+	−	−	M. fortuitum
					
III	7	−	−	−	Non-mycobacteria^*a*^

a Escherichia coli, Klebsiella pneumonia, Staphylococcus aureus, Pseudomonas aeruginosa, Candida kruseii, Acinetobacter baumannii, Nocardia asteroides.

### Culture and direct microscopic observation.

All 169 samples were tested by mycobacterial staining/culture assays, of which 43 were positive in mycobacterial growth and 33 were positive in acid-fast staining assays (Table 2). Among 43 samples with positive mycobacterial growth, 34 were identified as M. tuberculosis, 5 as NTM species including 3 M. intracellulare and 2 M. chelonae*-abscessus*, and 4 as pathogens other than Mycobacterium. These NTM species were also detected by Ziehl-Neelsen (ZN) staining.

**TABLE 2:** Comparison of aptamer MA1-39 with conventional procedures for M. tuberculosis detection in 169 clinical sputum samples^*a*^

Group	Smear/culture		Test						Organism	Sample origin
			16sRNA		IS6110		Aptamer			
			P	N	P	N	P	N		
I	P/P	24	24	0	22	2	21	3	M. tuberculosis	TB
	P/N	4	4	0	4	0	3	1	M. tuberculosis	TB
	N/P	10	10	0	10	0	9	1	M. tuberculosis	TB
II	N/N	48	23	25	15	33	9	39	ND^*c*^	TB
III	P/P	1	1	0	0	1	0	1	M. intracellulare	TB
IV	P/P	2	2	0	0	2	0	2	M. chelonae-*abscessus*	Non-TB
		2	2	0	0	2	0	2	M. intracellulare	Non-TB
V	N/P	4^*b*^	0	4	0	4	2	2	ND	Non-TB
VI	N/N	6^*c*^	0	6	0	6	6	0	ND	Non-TB
VII	N/N	68	0	68	0	68	0	68	ND	Non-TB
										
Total		169	66	103	51	118	50	119		

a P, positive; N, negative, TB, tuberculosis; ND, not determined.

b Possible false-positive result by culture.

c Possible false-positive result by aptamer test.

### Comparison of aptamer detection in sputum specimens with clinical and bacteriological data.

A total of 169 samples were tested by MA1-39 fluorescence microscopy (Fig. 2B), conventional bacteriological tests, and IS6110-PCR amplification. Based on clinical data, a definitive diagnosis of TB was made in groups I, II, and III. In group I, 24 samples were positive for both smear and culture; the positive rates of IS6110 and MA1-39 detection in these samples were 91.7% (22/24) and 87.5% (21/24), respectively. The remaining 14 samples in group I were either smear-positive but culture-negative (*n* = 4) or smear-negative but culture-positive (*n* = 10); the positive rates of IS6110 and MA1-39 detection were 100% (14/14) and 85.7% (12/14) in these samples, respectively. In group II, 48 samples were negative for both smear and culture, and the positive rates of MA1-39 (18.8%, 9/48) and IS6110-PCR detection (31.25%, 15/48) were not high in these samples. The results are shown in Table 2.

Group III included one sample from a patient showing clinical manifestations of a mycobacterial infection (Table 2). This sample was positive for smear and culture, but negative for MA1-39 and IS6110 detection, and was diagnosed as NTM infection by 16S rRNA sequencing. Finally, of the 82 samples from non-tuberculosis patients, 82.9% (68/82) were negative in smear/culture and for MA1-39 and IS6110 detection (group VII, Table 2). However, of the remaining 14 specimens, 4 were positive for smear and culture but negative for the aptamer and IS6110 tests, and these were identified as NTM infections based on 16S rRNA sequencing (group IV, Table 2); 4 were positive for culture but negative for smear, and these were detected as pathogenic bacteria other than mycobacterium by IS6110 and 16SrRNA amplification (group V, Table 2); and 6 were positive for aptamer but negative for both culture**/**smear and PCR-amplification (group VI, Table 2).

### Performance characteristics.

To determine true-positive and true-negative groups for TB infection, a comparison of both clinical and bacteriological criteria and patient history was performed to resolve the discrepant analysis. The results showed that 86 specimens form groups I and II were defined as true positives for TB infection. Among 83 specimens defined as true negatives for TB, 78 were from groups V, VI and VII and 5 specimens were from groups III and IV (3 M. intracellulare and 2 M. chelonae*-abscessus*). The sensitivity and specificity of aptamer, IS6110, and culture/staining assays were assessed in comparison with the clinical assessments of the patients. These results are shown in Table 3.

**TABLE 3:** Aptamer MA1-39 results compared with culture and staining results and clinical assessment of patients^*a*^

Test and result	No. of specimens		Sensitivity (%)	Specificity (%)	PPV (%)	NPV (%)
	Positive	Negative				
Stain						
Positive	28	5	32.6	94	84.9	57.4
Negative	58	78				
Culture						
Positive	34	9	39.5	89.2	79.1	58.7
Negative	52	74				
Aptamer						
Positive	42	8	48.8	90.4	84	63
Negative	44	75				
IS6110-PCR						
Positive	51	0	59.3	100	100	70.3
Negative	35	83				

a PPV, positive predictive value; NPV, negative predictive value.

Compared with traditional acid-fast staining microscopy, aptamer-fluorescence microscopy showed almost no significant difference in specificity (90.4% versus 94%, *P* = 0.0593). However, the positive detection rate of aptamers for TB in clinical sputum specimens was higher than that of acid-fast staining (48.8% versus 32.6%, *P* < 0.0001). The positive and negative predictive values (PPV and NPV) for the staining, culture, aptamer, and IS6110 tests were 84.9% and 57.4%, 79.1% and 58.7%, 84% and 63%, and 100% and 70.3%, respectively. The aptamer test showed the same PPV as staining (84.89% versus 84%, *P* = 0.5158), but a higher NPV (57.4% versus 63%, *P* = 0.0010).

## DISCUSSION

Microbiological detection of TB is critical because it allows people to be correctly diagnosed and ensures that the most effective treatments regimens can be selected as early as possible. Of the 4.8 million people diagnosed with pulmonary TB worldwide in 2020, 59% were confirmed by bacteriological tests (1). Conventional bacteriological diagnosis of TB mainly relies on acid-fast staining microscopy of specimens, combined with isolation and culture of the bacilli to specifically detect mycobacteria. Rapid molecular testting has been recommended for use as an initial TB diagnostic test by the WHO, but its use is still far too limited. Of the 5.8 million people newly diagnosed with TB in 2020, only 1.9 million (33%) were diagnosed using molecular testing as the initial test (1). Sputum-smear microscopy is simple, fast, and inexpensive, and has become one of the important methods for clinical diagnosis of TB. In fact, this microscopy method is still used in many laboratories, especially those in low-income countries (5, 21, 22). However, due to the low sensitivity of smear microscopy, which requires >10,000 bacilli per mL of sputum, the positive detection rate of TB in clinical specimens is not high (23). Thus, there is an urgent need to improve smear microscopy to solve these current diagnostic problems.

To date, numerous physical and chemical sputum-processing methods, such as centrifugation, *N*-acetylcysteine-sodium hydroxide, bleach, ammonium sulfate, and chitin, have been investigated to improve smear microscopy (24, 25). Another recent development is that fluorescence microscopy has been used in smear microscopy (10, 25). A systematic review reported that fluorescence microscopy could increase the sensitivity of smear microscopy compared to the conventional acid-fast light microscopy mostly used in poorer countries (10% increase) (19). Fluorescence microscopy may allow better quality microscopy to be accomplished with the same human resources as used for acid-fast staining microscopy (18) and has been recommended as an alternative to light microscopy (18).

In addition, the accurate recognition of pathogenic bacteria is also beneficial to improve the performance of smear microscopy. Immunofluorescence microscopy with an anti-MTB antibody as the recognition element has been demonstrated as having potential application for rapid detection of MTB in clinical samples (10). Recently, considerable attention has been given to aptamers, which are sometimes called “chemical antibodies” because of their similarities to biological receptors. Compared to traditional antibodies, aptamers, developed by SELEX, have unique features, such as low-cost production that does not require a living system, high chemical stability and affinity, easy chemical modification, and low-level immunogenicity and toxicity (14, 15). Currently, novel fluorescent detectors based on aptamers have shown clinical application potential for the rapid detection of pathogenic microorganisms (15, 16).

In our previous study, we also used whole-cell SELEX to develop MTB-specific aptamers (17). In this study, we tried to use aptamer fluorescence microscopy to directly detect MTB in complex sputum specimens to evaluate its feasibility for the diagnosis of pulmonary TB. To improve the power of this test for TB diagnosis, we first modified the original aptamer MA1 and obtained three derivative aptamers: MA1-23 with only one pocket structure, MA1-39 with one pocket-stem-loop-structure, and MA1-55 with most of the original structure. The results showed that MA1-39 had significant binding-reactivity with H37Rv, and FITC-labeled MA1-39 fluorescence microscopy observation effectively discriminated the MTB strain from the control strains.

Subsequently, we evaluated the diagnostic efficiency of the MA1-39 fluorescence microscopy method in clinical sputum specimens. Among the three bacteriological assays, traditional acid-fast staining microscopy showed the highest specificity (94%), but the lowest sensitivity (32.6%), which was significantly lower than the sensitivity of aptamer detection (48.8%). After resolving the discrepant analysis by comparing the aptamer test in sputum specimens with clinical and bacteriological data, the aptamer test showed the same positive predictive value as acid-fast staining and the highest negative predictive value in three assays. These results indicated that aptamer fluorescence microscopy observation was sensitive. The use of aptamer probes significantly increased the positive detection rate of TB in clinical sputum specimens.

Moreover, efficient digestion of sputum is critical for aptamer detection. If the bacteria in the sputum cannot be effectively released, the aptamer cannot effectively bind to the target bacteria. In addition, complex sputum samples might contain some inhibitors that affect the binding of aptamers to the target bacteria, and repeated washing might also result in the loss of some bacteria. Therefore, the current aptamer assays had some limitations and failed to estimate the number of bacteria in complex sputum samples as effectively as ZN microscopy. Additional studies are needed to improve aptamer assays. Nevertheless, our study results are sufficient to demonstrate that aptamer fluorescence microscopy, with its higher sensitivity compared to conventional ZN microscopy, offers a feasible alternative for microbiological diagnosis of TB. Once fully developed, methods based on MTB-specific aptamers may be effective tools for early diagnosis of pulmonary TB.

Rapid and accurate detection of MTB in clinical samples is important for TB control. Many advanced technologies have been used to enhance or improve diagnostic capacity and efficiency. At present, molecular tests, such as the Xpert MTB/RIF assay, provide rapid and specific detection systems for MTB in clinical specimens. However, the sensitivity of molecular tests is still far from ideal, and their use as part of a series of tests is not cost-effective (2, 3, 26, 27). Molecular tests increase diagnostic efficiency and certainty, but they neither replace nor reduce the need for smears and cultures at this time (2, 26). In the foreseeable future, microscopy may still be the primary and most common test method for microbiological diagnosis of TB in many laboratories in resource-limited countries (3, 23, 27). Improvements in clinical sputum collection, sputum processing methods, and detection technologies can further enhance the clinical application value of this ancient diagnostic method. Since aptamer fluorescence microscopy can produce rapid results and is also more cost-effective than molecular techniques, it may be a feasible and useful method in resource-limited settings.

## MATERIALS AND METHODS

### Ethics statement.

All human participants were treated in accordance with the Declaration of Helsinki. This study was ethically approved by the Nantong Sixth People’s Hospital Ethics Committee (permit number: NTLYLL2021002).

### Aptamer.

To improve diagnostic efficiency, we designed three derivative aptamers based on the secondary structure analysis of aptamer MA1, obtained from our previous study (17). The new derivative aptamers had different secondary structures: a 23-nt DNA aptamer with only a pocket structure (MA1-23), a 39-nt DNA aptamer with a pocket-stem-loop-structure (MA1-39), and a 55-nt DNA aptamer with a similar structure to the original aptamer MA1 (MA1-55). All these aptamers were synthesized and labeled with biotin or fluorescein isothiocyanate by Sangon Biotech Co., Ltd. (Shanghai, China) with high-pressure liquid chromatography purification. Oligonucleotides were dissolved in sterile double-distilled water (ddH_2_O) and DNA concentrations were assayed using an Infinite M200 PRO NanoQuant Microplate Reader.

The binding affinities of the derivative aptamers against H37Rv were tested in an enzyme-linked immunosorbent assay, as described in our previous study (17). Briefly, H37Rv were coated on the surface of the plate, biotin-labeled aptamer was used as the detection molecule, and streptavidin-horseradish peroxidase-substrate system was added for detection. The optical density (OD) at 450 nm was determined by a spectrophotometer (Multiskan MK3; Thermo Fisher Scientific, Waltham, MA). The difference of binding affinity between derivative aptamers was performed by two-tailed unpaired *t* tests using GraphPad Prism 5.0 software. Statistical significance was defined as *P* < 0.05.

### Tested strains.

A total of 56 test strains were prepared to evaluate binding reactivities of the aptamers derived from MA1 against the MTB strain, including the reference strain H37Rv (ATCC 27294), 30 MTB isolates, 12 NTM species (M. intracellulare [ATCC 13950], M. abscessus [ATCC 19977], M. avium [ATCC 25291], M. kansasii [ATCCl2478], M. gordonae [ATCC 14470], *M. gilvum* [ATCC 43909], *M. aurum* [ATCC 23366], M. marinum [ATCC 927], M. smegmatis [ATCC 19420], *M. aichiense* [ATCC 27280], *M. phlei* [ATCC 11758], M. fortuitum [ATCC 6481]) and 6 NTM isolates (2 from M. intracellulare, 3 from M. abscessus, and 1 from M. kansasii; these species are the common causative organisms of NTM pulmonary infection), and 7 non-mycobacterial isolates from common pathogenic bacteria of other pulmonary infections, including E. coli, Klebsiella pneumonia, Staphylococcus aureus, Pseudomonas aeruginosa, Candida kruseii, Acinetobacter baumannii, and Nocardia asteroides. All tested strains and isolates are listed in Table 1. The genomic DNA from all tested strains and isolates were amplified by mycobacterial 16S rRNA genes (28, 29) and IS6110 specific for the MTB complex (30, 31) as a control. All isolates were isolated by Shanghai Pulmonary Hospital. Mycobacterial tested strains and isolates were prepared as described in our previous study (17). In brief, all mycobacteria were grown in Middlebrook 7H9 medium. Cells were collected by centrifugation and washed with phosphate-buffered saline (PBS). Amounts of bacteria were evaluated using a PhoenixSpec nephelometer (BD Biosciences). Cells were then sterilized at 80°C for 30 min and stored at −20°C. Seven non-mycobacterial bacteria isolates were scraped from the blood plate and prepared similarly to the mycobacteria.

### Characteristics of the experiment participants.

A total of 169 cases of clinical sputum specimens were collected by the Nantong Sixth People’s Hospital (Nantong, Jiangsu, China) to test the clinical applicability of aptamers in TB diagnosis. These included 87 TB patients (68 males and 19 females, aged 15 to 97 years, average age of 47.59 ± 2.368 years) and 82 non-TB controls (58 males and 24 females, aged 6 to 85 years, average age of 52.46 ± 2.392 years), including 13 healthy volunteers and 69 non-TB patients. Statistical analysis was performed by two-tailed unpaired *t* tests using GraphPad Prism 5.0 software. There were no significant differences in average age and gender between the two groups (*P* > 0.05).

A definite diagnosis of TB was made according to criteria by the Chinese Antituberculosis Association, which mainly relying on TB symptoms and signs, identification of MTB in a clinical sample, TB lesions detected by computed tomography scan or chest X-ray, and symptom relief from anti-TB treatment. The 69 non-tuberculosis patients were positive for pneumonia (17 cases), lung cancer (25 cases), lung abscess (15 cases) and other conditions (12 cases).

### Sputum samples and preparation.

Sputum samples were digested by standard protocols with *N*-acetyl-l-cysteine–2% NaOH for 15 ~ 20 min (32). The digested samples were mixed with 40 mL of 0.067 mol/L PBS and then centrifuged at 3,000 × *g* for 20 to 30 min. The resulting sediment was resuspended in 500 μL PBS. Aliquots of the resuspended sediments of sputum were frozen at −20°C until they were processed for bacteriological or molecular tests.

### Culture and direct microscopy.

All digested/concentrated sputum samples were used to prepare mycobacterial cultures by inoculating onto conventional Loewenstein-Jensen slants, and incubated at 37°C for 8 weeks, with weekly observation for the presence of mycobacterial colonies (33). Positive colonies were confirmed by Ziehl-Neelsen staining and then identified as MTB or other mycobacteria species using both IS6110-PCR amplification and 16SrRNA sequencing (34). A 99% identity was used to define a specific species by comparing the 570 bp of the 16SrRNA sequence to those deposited in the GenBank database (http://www.ncbi.nlm.nih.gov/BLAST/). Concentrated sputum from the same specimen was also stained using the ZN method and performed for smear examination by microscopy (33).

### Aptamer observation.

Aptamers with FITC were used to prepare for fluorescence microscopy observation. Denatured aptamers, obtained by heat treatment at 94°C for 5 min and then room temperature for 15 min, were incubated with the tested strains/isolates (2 × 10^7^ CFU) or with digested/concentrated sputum samples in 500 μL PBS at 37°C for 40 min with gentle rotation, and then centrifuged at 12,000 × *g* for 5 min. After washing twice with 1,000 μL PBS, the resulting sediment was resuspended in 20 μL ddH_2_O and smeared on microscopic slides. The full field of view for every slide was observed by a fluorescence microscope (Leica DMi 8, LEICA, Germany) with a ×40 magnification objective. Fluorescence intensities were assessed by computer-assisted analysis using Image J (v1.8.0, National Institutes of Health, Bethesda, MD).

### Statistical analysis.

We calculated the sensitivity, specificity, positive predictive value, and negative predictive value. All statistical analyses were performed using GraphPad Prism 5.0 software. Two-tailed unpaired *t* tests were used to analyze differences of sensitivity, specificity, PPV, and NPV between the traditional acid-fast staining microscopy and aptamer-fluorescence microscopy methods. The significance level was set at *P* < 0.05.
